# Supplementary material for: Some novel concepts of interval-valued q-rung orthopair fuzzy graphs and computational framework of fuzzy air conditioning system
Source: PLoS One. 2025 Apr 25;20(4):e0317676. doi: 10.1371/journal.pone.0317676 (PMC12029560; doi:10.1371/journal.pone.0317676)
Supplement: Attachment — Submitted filename: Reviewer-2 comments with responses.docx [file pone.0317676.s001.docx]

**Reviewer-2 Comments with Responses:**

**Manuscript ID: PONE-D-24-37248**

**Title: Some Novel Concepts of Interval-valued q-rung orthopair Fuzzy Graphs and Computational Framework of Fuzzy Air Conditioning System**

We appreciate the reviewer for their time and precious comments. No doubt, after endorsing these comments our article would become more effective. The point by point response of the reviewer comments are as follows.

**Reviewer #2:**

Dear authors,

(1) The abstract lacks clarity, focus, and a clear contribution to the field. First, the introduction of "fuzzy sets" is vague and poorly contextualized; it reads more like a generic description rather than an insightful introduction to the problem. The mention of interval-valued q-rung orthopair fuzzy sets (q-ROFSs) is abrupt and doesn't build on the previous information, leaving the reader confused about how it significantly improves over existing methods. The abstract does not clearly justify why q-ROFSs are a "more flexible tool," which is a critical oversight in demonstrating the novelty of the study. Furthermore, the objectives of the study are presented in a fragmented and confusing manner. The transition from introducing fuzzy graphs to the application in a fuzzy air conditioning system (FACS) lacks logical flow, making it hard to follow the progression of ideas. The abstract feels like a collection of loosely related concepts that are not tied together well. The second objective, providing a computational framework for the FACS, is extremely underdeveloped. There's no clear explanation of how this framework works, its unique contributions, or how it advances the field. Phrases like "provide evidence through comparative study" are vague and unsubstantiated, as the abstract doesn't clarify what exactly was compared or what the benchmark for success was? the abstract lacks any mention of managerial, practical, which would make the research more valuable and applicable. The overall tone is overly technical without being informative, making it inaccessible to a broader audience. The writing is overly complex without justification, leading to unnecessary confusion. This abstract needs a complete overhaul to ensure clear articulation of the problem, methodology, and the real-world relevance of the findings.

**Response.** Thank you very much for the reviewer for his careful revision and valuable suggestions. We must describe the incorporations in the following points:

i. We have revised the abstract section, properly. In this regard, we have elaborated the significance of study on q-ROFSs compared to other generalizations of FSs.
ii. We have updated the section of motivation and novelty of our work.
iii. We also updated the application section in order to make a logical flow. In this regard, we relate a FACS with temperature sensors, instead.
iv. As per reviewer’s suggestion, we have provided a pseudocode and algorithm which indicate how this computational framework works.
v. We have also rectified the section of comparative analysis by elaborating the worthiness of our study through examples. Moreover, we have also added a subsection “Discussions”, in which we compare our proposed study by existing ones.
(2) Incredibly, this manuscript lacks a dedicated section for the literature review, which raises the question: wouldn’t it be more appropriate to include a thorough review of relevant research? This would provide readers with a better understanding of the existing knowledge base and where your work fits in.

**Response.** As per reviewer’s suggestion, we have added a section “Literature review” in which we have revised the relevant study and clearly highlighted the importance of our work.

(3) Furthermore, there is no discussion section, which is crucial for comparing your findings with those of other studies. Readers are interested in seeing a real discussion between your results and previous research.

**Response.** We have added a discussion as a subsection of a Section 6 which includes the comparison between our findings to the existing studies.

Additionally, the conclusion is extremely brief. It could benefit from being divided into distinct sub-sections, such as theoretical contribution, managerial implications, and practical applications. As a decision-maker in a public or private sector organization, how would this research help me? Please clearly articulate these aspects. Moreover, include a section titled "Implications" that addresses the potential impact of this research on the scientific community in the coming months and years.

**Response.** As per reviewer’s suggestion, we have revised the conclusion section. We have elaborated the notions that we have introduced. Moreover, we have also provided a theoretical contributions, managerial implications and practical applications of our study in the subsection " Implications of our proposed study”.

(4) A section on research limitations and future recommendations is also necessary for a well-rounded paper.

**Response.** As per reviewer’s suggestion, we have added a section about the research limitations and future directions of our proposed study.

While the paper currently has structural and methodological shortcomings, I am inclined to give it a chance due to its contribution to advancing fuzzy concepts. With substantial revisions, it has the potential to meet the required standards.
